# Understanding the Formation of Apoferritin Amyloid Fibrils

**DOI:** 10.1021/acs.biomac.1c00176

**Published:** 2021-04-06

**Authors:** Rocío Jurado, Jozef Adamcik, Antoni Sánchez-Ferrer, Sreenath Bolisetty, Raffaele Mezzenga, Natividad Gálvez

**Affiliations:** † Department of Inorganic Chemistry, University of Granada, 18071 Granada, Spain; ‡ Department of Health Sciences and Technology, ETH Zürich, 8092 Zürich, Switzerland; § Department of Materials, ETH Zürich, 8093 Zürich, Switzerland

## Abstract

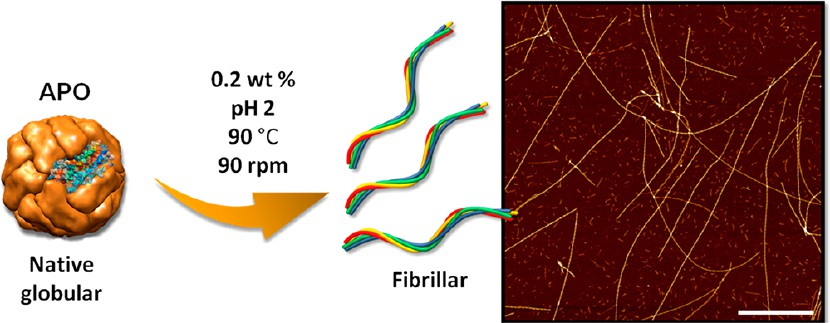

We present the optimization of experimental conditions to yield long, rigid apoferritin protein amyloid fibrils, as well as the corresponding fibrillation pathway. Fibril growth kinetics was followed using atomic force microscopy (AFM), transmission electron microscopy (TEM), dynamic light scattering (DLS), circular dichroism (CD), fourier-transform infrared spectroscopy (FTIR), and sodium dodecyl sulfate polyacrylamide gel electrophoresis (SDS-PAGE). Among the morphologies identified, we show that the conditions result in small aggregates, as well as medium and long fibrils. Extended incubation times led to progressive unfolding and hydrolysis of the proteins into very short peptide fragments. AFM, SDS-PAGE, and CD support a universal common fibrillation mechanism in which hydrolyzed fragments play the central role. These collective results provide convincing evidence that protein unfolding and complete hydrolysis of the proteins into very short peptide sequences are essential for the formation of the final apoferritin amyloid-like fibrils.

## Introduction

Amyloid aggregates are used by nature in numerous and creative ways, ranging from bacteria to humans. Functional amyloid materials include natural as well as artificial materials ranging from drug delivery systems to components in active materials and sensors.^1−4^ Such structures possess many biological roles including catalytic functions, mammalian skin pigmentation or the peptide/protein hormones in secretory granules of the endocrine system. Thus, functional amyloids contribute to normal cell and tissue physiology.^3,5^ Additionally, amyloid fibrils are pathological features of a number of harmful neurodegenerative diseases.^6−10^

Amyloid structures originate from a wide range of soluble peptides and proteins, wherein peptide or protein monomers spontaneously self-associate into small oligomers, then into supramolecular aggregates, and finally, they form fibrillar structures.^2,11^ Peptides and globular proteins are known to possess an intrinsic tendency to convert from their native functional states into insoluble amyloid fibrils.^12,13^ It is generally agreed that the process of fibril formation starts from a nucleation site or seed comprising partially unfolded proteins.^11,14,15^ Native-state globular proteins, as opposed to smaller pathological peptides, possess a condensed, rigid structure, so their fibrillation occurs from the destabilization of the native structure into partially unfolded conformations via substantial changes in environmental conditions (mainly temperature and pH), which are usually extremely denaturing.

The iron-regulatory protein ferritin, plays an essential metabolic role in practically all life forms. Apoferritin (APO) is the iron-free ferritin protein, which possesses a globular structure composed of 24 polypeptide subunits or chains (*M*_r_ ∼ 480 kDa) that store iron in the form of an iron oxide nanoparticle, mainly ferrihydrite.^12^ The APO organic capsid mainly results from the self-assembly of two polypeptide chains: the L (light, 20 kDa) and H (heavy, 21 kDa) subunits. The self-assembly of the H- and L-chains into the 24-mer APO capsid originates a variety of different ferritin molecules whose H:L composition is genetically controlled in each tissue and organ. The H:L ratio in all organs and tissues is determined by their functionality.

We have recently reported that the globular horse spleen apoferritin protein (L-rich APO = L_21_H_3_) can undergo fibrillization at temperatures ranging from 50 to 80 °C and pH values of 2 to 5. We have already confirmed the amyloid nature of these fibrils and that their chirality depends on the original peptide sequence, either L- or H-subunits.^16^ In the present work, we study the chemical conditions necessary to optimize fibrillation, as well as the kinetics of the genesis and development of L-rich APO amyloid fibrils, hereafter APO, by investigating their structure–time evolution using various analytical (macro) and structural (micro) techniques. The combination of these techniques identifies different individual stages during fibrillation, in agreement with similar studies of different protein amyloid fibrils.^17−19^

We decided to study APO fibril formation for two reasons. First, alterations in ferritin functions have been associated with common iron-related diseases such as hemochromatosis and anemia,^20^ coupled with the increasing recognition of ferritin’s crucial role in some neurological pathologies such as Parkinson’s and Alzheimer’s disease.^21^ Second, our interest also lies in the possibility of engineering and integrating different properties into fibrils, so that we may create functional materials with new or improved properties.

## Materials and Methods

### Chemical Parameters Controlling APO Fibrillation

Horse spleen apoferritin protein was purchased from Sigma-Aldrich. Protein solutions were adjusted to pH 2 (1 M HCl in Milli-Q water) before heating (90 °C in hermetically sealed glass tubes). To optimize the protein concentration, four aliquots of APO were prepared at concentrations of 0.05, 0.1, 0.2, and 0.4 wt %, then heated on a stirrer hot plate for 9 h and at 90 rpm. To optimize incubation time and stirring rate, four aliquots of 0.1 wt % APO were prepared and heated for 9 or 24 h and at 90 or 220 rpm. These aliquots were then labeled according to incubation time, stored at 4 °C, and used for structural analysis without further manipulation.

### APO Aggregation Kinetics

Protein solutions (0.2 wt %) were adjusted to pH 2 (1 M HCl in Milli-Q water) then heated (90 °C in hermetically sealed glass tubes). Aliquots of the samples were collected at 0 min, 5 min, 15 min, 30 min, 45 min, 1, 3, 5, 9, and 24 h after starting incubation and quenched immediately in an ice–water bath to arrest the conversion of monomers into fibrils. These aliquots were then labeled according to incubation time, stored at 4 °C, and used for structural analysis without further manipulation. APO aggregation kinetics was repeated for at least three times and different samples were used in the following experiments.

### Transmission Electron Microscopy (TEM) Imaging

All samples were prepared by placing a drop onto a carbon-coated 200 mesh Cu grid. Electron micrographs were taken with a LIBRA 120 PLUS microscope operating at 120 keV.

### Atomic Force Microscopy (AFM) Imaging

AFM experiments were carried out on a Multimode 8 Scanning Probe Microscope (Bruker, U.S.A.) covered with an acoustic hood to minimize vibrational noise. A droplet of the different aliquots was deposited onto freshly cleaved mica, incubated for 2 min, rinsed with Milli-Q water, and dried under nitrogen. The AFM was operated in tapping mode under ambient conditions using commercial silicon nitride cantilevers (Bruker, U.S.A.) at a vibration frequency of 150 kHz. Images were simply flattened using Nanoscope 8.1 software, and no further image processing was carried out. The resulting images were used for the statistical analysis. AFM images were traced using FiberApp software.^22^

### Statistical Analysis

AFM images were traced using FiberApp software.^22^ The statistical analysis of the different concentrated samples was conducted systematically on more than 500 aggregates for each concentration reported. For fibrils incubated at different times and stirring rates, the statistical analysis was conducted systematically on more than 160 aggregates for each sample reported. For the kinetics experiment, the statistical analysis of the fibrils size, length and persistence length was conducted systematically on more than 550 aggregates for each incubation time reported.

### Dynamic Light Scattering (DLS)

The APO fibril intermediates formed at different incubation times were studied by light scattering using a LS Instruments machine equipped with a He–Ne laser emitting a polarized light beam at a wavelength of 632.8 nm. DLS measurements were performed at a fixed angle of 90° by averaging 3 runs of 600 s each. The time correlation function (TCF) of the scattered intensity was analyzed using the CONTIN method.

### Sodium Dodecyl Sulfate Polyacrylamide Gel Electrophoresis (SDS PAGE)

For each sample, 12 μL of 0.2 wt % aliquots was mixed with 3 μL of dithiothreitol (DTT) and 15 μL of XT sample buffer 4× (Bio Rad Laboratories). The solutions were then heated for 10 min at 90 °C. The 30 μL solutions and molecular weight (*M*_w_) markers (PageRuler Unstained Protein Ladder) were then loaded on 19% stacking gel buffer and separated at 100 V (10 min) and 200 V (40 min). The gel was collected and stained for 30 min in a Coomassie blue dye solution (0.1% Coomassie blue R-250, 40% methanol, 10% acetic acid in water) in a closed container with continuous agitation. This was followed by several destaining steps with a destaining solution (40% methanol, 10% acetic acid) with continuous agitation. SDS-PAGE experiments were repeated three times.

### Thioflavin T Analysis

The formation of amyloid aggregates was detected as increased ThT fluorescence intensity. Sample fluorescence was recorded after the addition of 25 μL of 0.1 wt % APO aliquots to 25 μL of 56 μM ThT solution at pH 2. The measurements were performed in quartz cuvettes using a Cary Eclipse fluorescence spectrophotometer. Each sample’s emission spectrum was recorded at 482 nm after excitation at 412 nm. The slits were adjusted to 20 and 20 nm for the excitation and emission, respectively.

### Far-UV Circular Dichroism (CD)

Samples containing long fibrils (3, 5, 9, and 24 h aliquots) were centrifuged three times using Amicon Ultra-4 centrifugal filter devices with a molecular weight cutoff (MWCO) of 50000 Da and the supernatant recovered. CD spectra of APO were recorded using a Jasco J-815 spectropolarimeter equipped with a Peltier-controlled cell holder. Spectra at 20 °C were collected using a precision quartz cell with a 2 mm path length from 190–260 nm, at a bandwidth of 1 nm and a scan speed of 50 nm/min. All spectra were recorded by diluting the incubated 0.2 wt % aliquot 10× in pH 2. Spectra were background subtracted, averaged over five scans, and smoothed using Spectra Manager software. The α-helical and β-sheet percentages were determined with CDPro software (CONTINLL, SELCON3, and CDSSTR analysis) based on a reference set of 56 proteins in the 190–240 nm wavelength range.

### Attenuated Total Reflectance Fourier-Transform Infrared Spectroscopy (ATR-FTIR)

FTIR spectra were obtained with a Varian 640 FTIR spectrometer equipped with a Specac Diamond ATR Golden Gate. Powdered samples were scanned over a range of 4000 to 400 cm^–1^ at a resolution of 4 cm^–1^ at room temperature and averaged over 64 scans. The α-helical and β-sheet percentages were determined by deconvolution and assignment of the resulting peaks to the corresponding secondary structure.

## Results and Discussion

### Chemical Parameters Controlling APO Fibrillation

**Figure 1:**
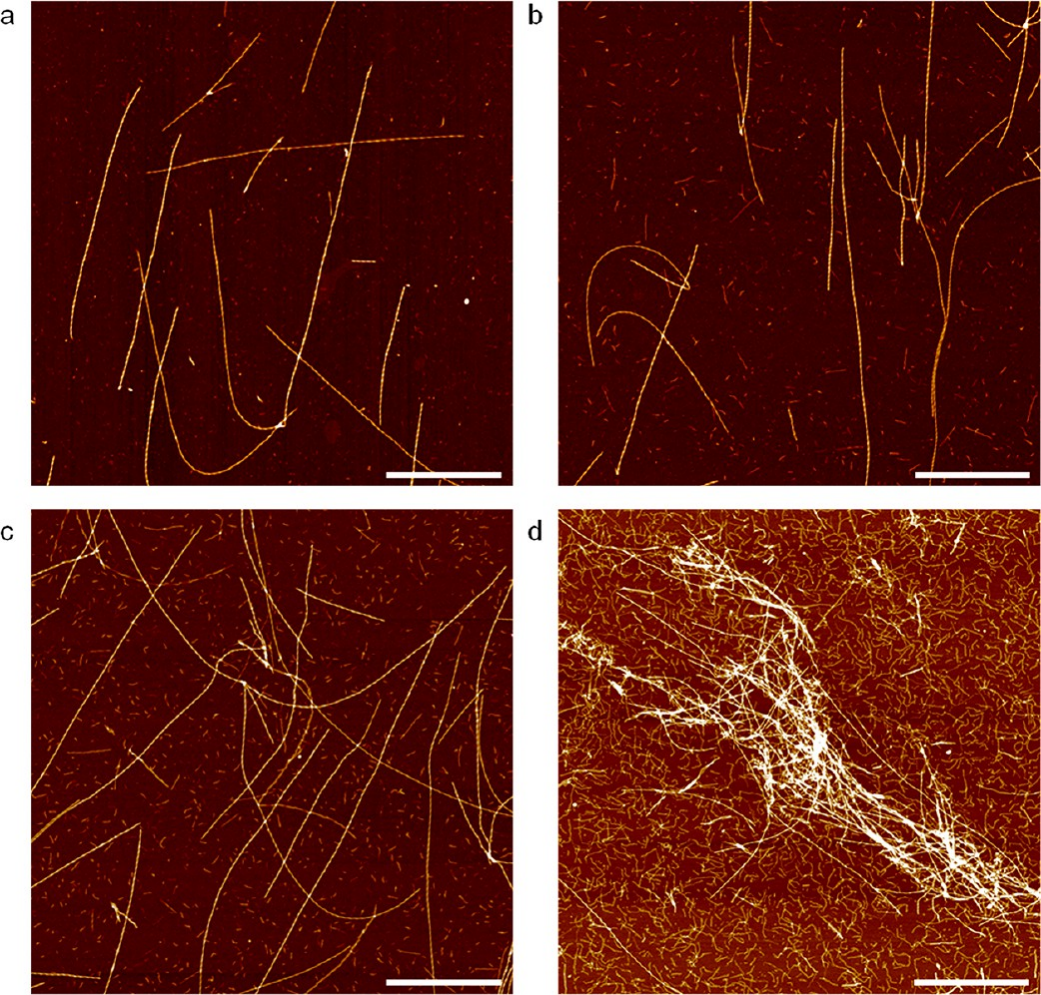
AFM images of (a) 0.05, (b) 0.1, (c) 0.2, and (d) 0.4 wt % APO after 9 h of incubation at 90 °C. Scale bars represent 1 μm.

The influence of protein concentration on APO fibril formation was studied by AFM. Figure S1 shows AFM and TEM images of native APO at physiological pH and pH 2. Protein concentrations between 0.05 and 0.2 wt % proved optimal in obtaining long, rigid fibrils of up to several microns in length (Figure 1) and with a persistence length in the micron range (Figure S2). However, a higher concentration, that is, 0.4 wt %, resulted in a greater number aggregated fibrils (Figure 1d), with a noticeable decrease in persistence length (Figure S2d). More representative images of the influence of protein concentration can be found in Figure S3.

**Figure 2:**
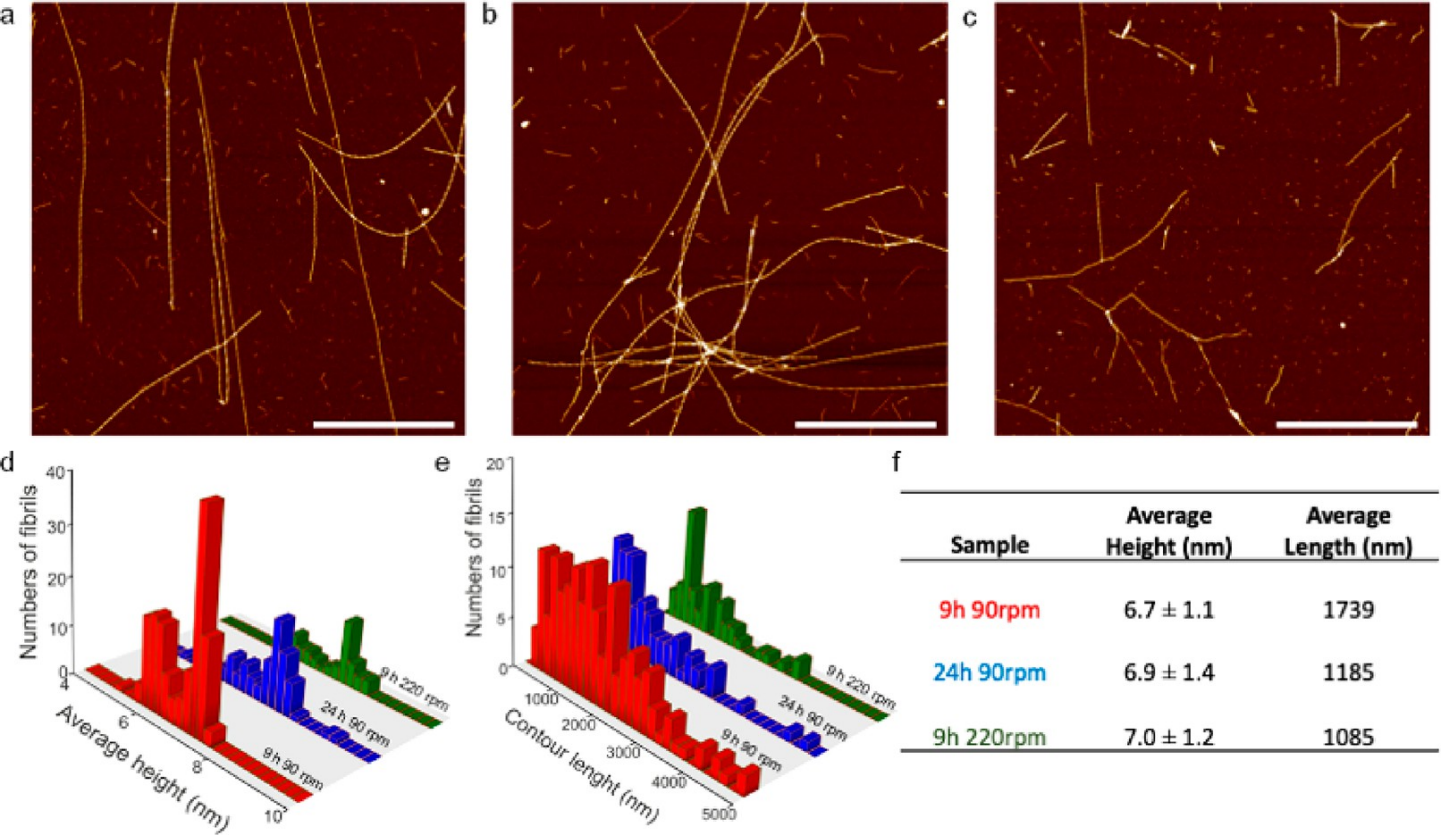
AFM images of APO heated at 90 °C: (a) after 9 h of incubation at 90 rpm, (b) after 24 h at 90 rpm, and (c) after 9 h at 220 rpm. Scale bars represent 1 μm. (d) Average height distribution, (e) contour length distribution, and (f) reported average height and length for the three samples.

The effects of incubation time and stirring rate on APO fibril formation were also studied by AFM (Figure 2), and the corresponding statistical analysis reported using FiberApp processing and tracking software.^23^ Therefore, we could make accurate measurements of the resulting fibrils’ contour length, average height, and persistence length. The protein fibrils formed after 24 h of incubation were shorter and more aggregated (blue distribution, average length = 1185 nm, Figures 2b,e,f and S4b) than those formed after 9 h (red distribution, average length = 1739 nm, Figures 2a,e,f and S4a) under the same stirring conditions. However, there were no significant differences in fibril height under 90 rpm, with averages of 6.7 and 7.0 nm for 9 and 24 h, respectively (Figure 2d,f). We attribute the decrease of length with time to the effect of stirring.

Similarly, fibrils formed at the higher stirring rate of 220 rpm suffered breakage and, therefore, yielded shorter lengths compared to the equivalent experiments at 90 rpm (Figures 2c,e,f, S4c, and S5).

Hence, after 9 h of incubation, shorter and more aggregated protein fibrils were formed by stirring at 220 rpm (green distribution, average length = 1085 nm, Figure 2e,f) in comparison with those produced at 90 rpm (average length = 1739 nm).

Fibrils formed after 24 h incubation at 220 rpm (Figure S5) had a shorter contour length of around 800 nm, as expected for the increase in both incubation time and stirring speed. For these experimental conditions an average height with two major population having 3.6 and 7 nm is observed (Figure S5b). We have recently reported that APO protein can form amyloid fibrils with different chirality and that that their chirality depends on the original peptide sequence in the protein, either L- or H-subunits. Thus, left-handed fibrils formed from H-subunits (H-APO) present an average height of 3.6 nm, thinner in comparison with right-handed fibrils formed from L-subunits (L-APO) with an average height of 7.0 nm. This can be at the origin of the wide distribution range of average height observed. Additionally, a process of unwind from a set of 2 fibrils could have happened.

The persistence length of the different APO fibrils was also extracted from the statistical analysis (Figure S6). This shows that APO fibrils heated for 9 h at 90 rpm had higher persistence lengths compared to those formed after the longer incubation time and higher stirring rate. This agrees with previous results, which yielded shorter, more aggregated fibrils upon increasing incubation time and stirring rate.^19^

Understanding the effect of small chemical changes on the final amyloid structure appears to be essential when studying amyloid formation. Thus, fibril formation depends on a number of experimental physicochemical conditions, primarily temperature, heating incubation time, and protein concentration. Stirring rate and pH also influence the final fibril structure. After considering all these parameters together, we have concluded that pH 2, incubation at 90 °C for 9 h, a protein concentration of 0.05–0.2 wt %, and a stirring rate of 90 rpm are the optimal synthetic conditions to form rigid, well-structured, wire-like fibrils with large persistence lengths from APO protein. These results are in consistent with previous protein fibril studies.^23,24^

### APO Aggregation Kinetics

We studied the growth kinetics of APO protein fibrils formed under optimal experimental conditions, that is, pH 2, 90 °C, 90 rpm, and a protein concentration of 0.2 wt %, by combining several techniques. Time-dependent intermediates of the evolving fibrils were collected by quenching aliquots of the samples at room temperature at specific incubation times (0, 5, 15, 30, and 45 min and 1, 3, 5, 9, and 24 h).

**Figure 3:**
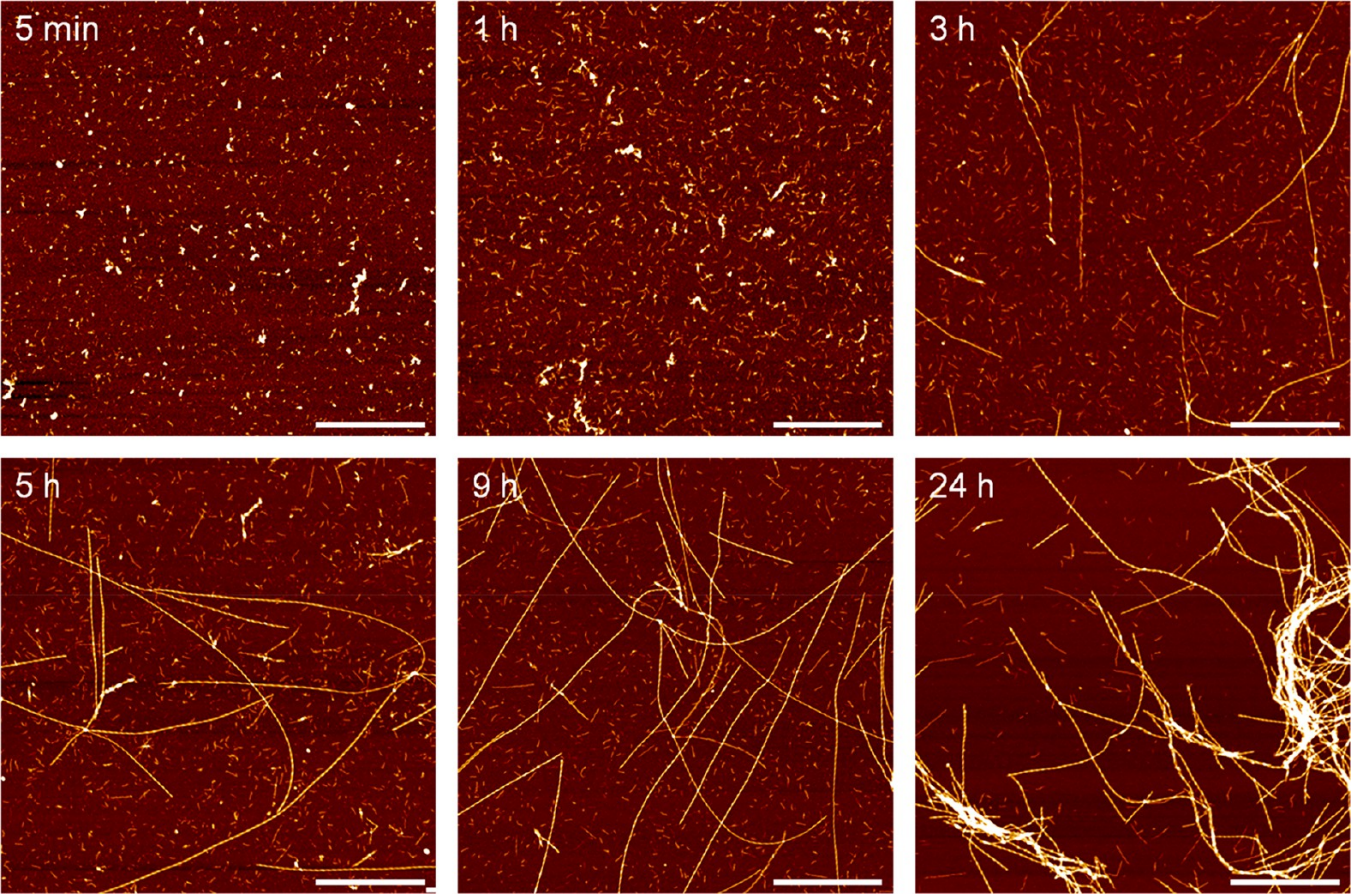
Real-space height AFM images of APO heated at 90 °C and pH 2 for different incubation times. Scale bars represent 1 μm.

The structural and morphology evolution of the APO fibrils was first characterized in detail using high resolution AFM. Figure 3 shows the height images of APO fibrils acquired after different incubation times.

**Figure 4:**
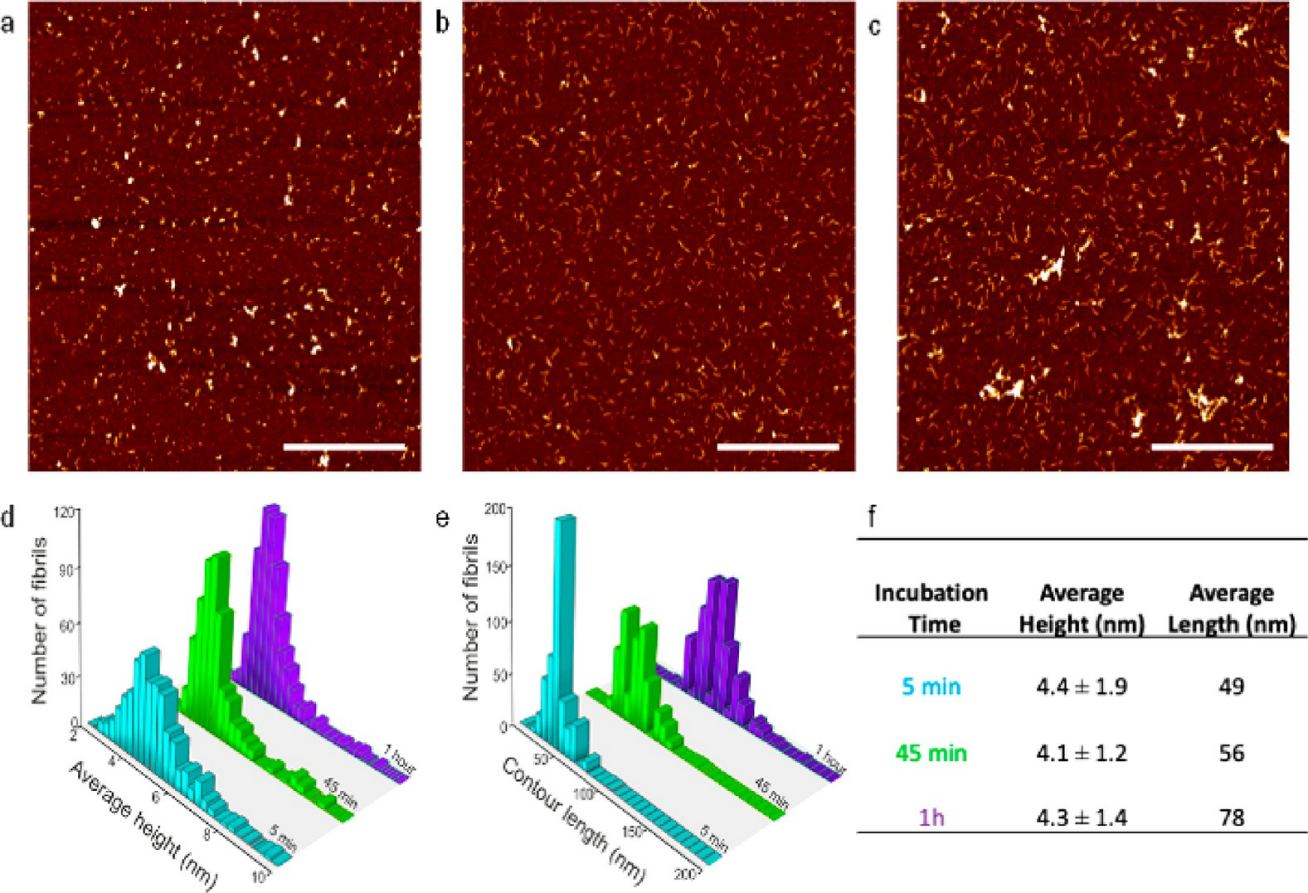
AFM images of APO heated at 90 °C after: (a) 5 min, (b) 45 min, and (c) 1 h of incubation. Scale bars represent 1 μm. (d) Average height distribution, (e) contour length distribution, and (f) reported average height and length for the three samples.

**Figure 5:**
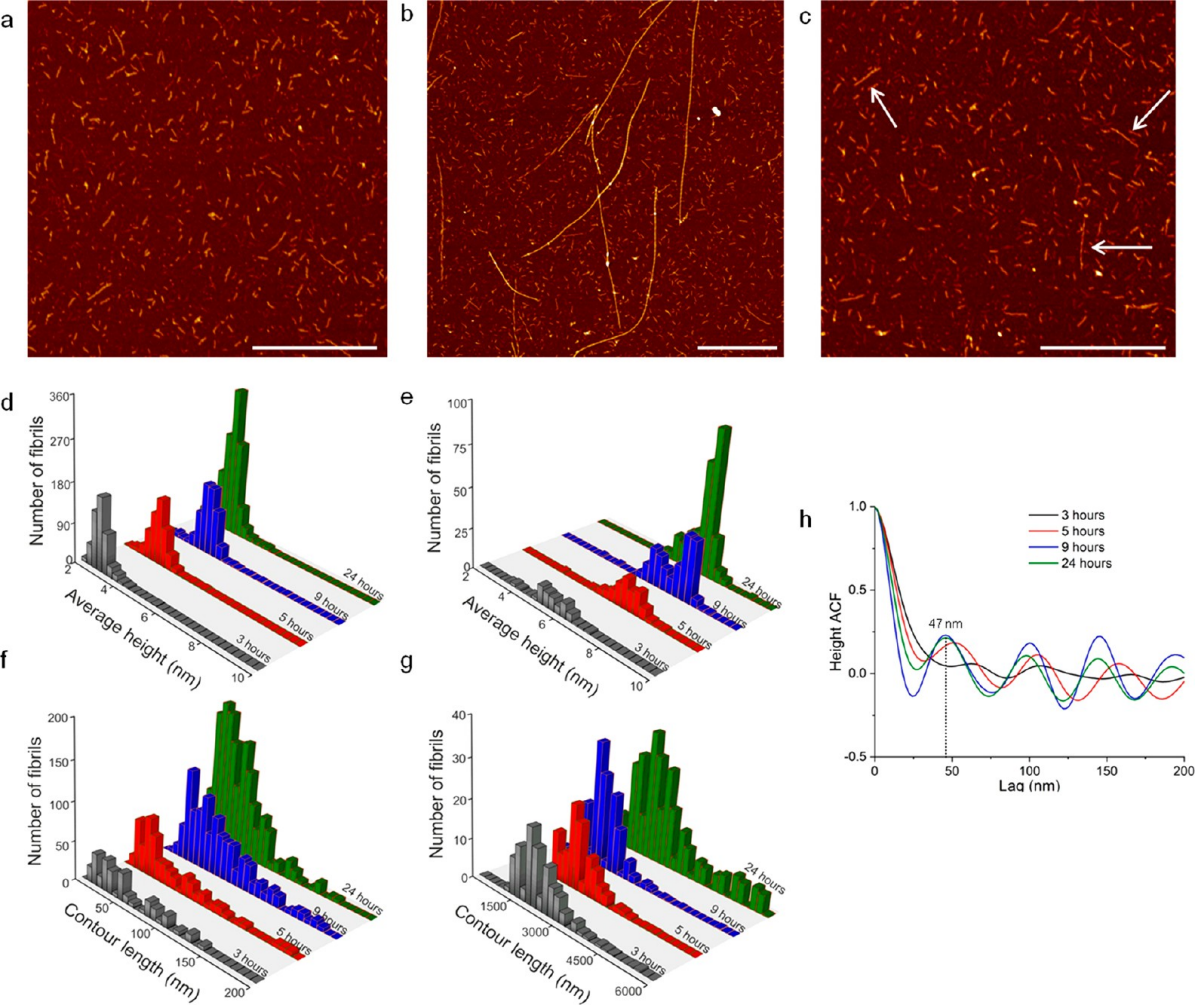
AFM images showing three APO populations after 3 h of incubation: (a) small aggregates, (b) long fibrils, and (c) medium-size fibrils (white arrows). Scale bars represent 1 μm. Average height distribution of (d) small aggregates and (e) long fibrils; contour length distribution of (f) small aggregates and (g) long fibrils after 3, 5, 9, and 24 h of incubation. (h) Pitch of long fibrils for the four incubation times estimated from AFM images using the autocorrelation function (ACF).

During the first hour, with no differences between 45’ and 1 h, there was an increase in the average contour length of the small protein aggregates or oligomers as a function of incubation time (Figures 4 and S7), but no long filaments/fibrils were observed. Long filaments first started to appear after 3 h of incubation time (Figure 3). These long rigid fibrils, developed in coexistence with small and medium aggregates (Figure 5a–c). The same three different populations were also observed after incubation times of 5, 9, and 24 h (Figure S8). These results indicate that the transition from APO oligomers into mature fibrils involves some critical transient steps.

The statistical analysis of the two extreme populations, that is, small aggregates and long fibrils, for the different incubation times is summarized in Figure 5 and Table S1. The analysis suggests that long fibrils grow between 3 and 9 h but they are shorter at 24 h. These results are consistent with an increase in fibril aggregation after longer incubation times, as shown previously by AFM (Figure 3) and in agreement with the behavior of other globular proteins.^19^ Long, rigid APO fibrils had an average maximum height centered at 6.6 ± 1.1 nm with a contour length of 1–4 μm (Figure 5e,g). Small aggregates, by contrast, presented an average height of 3.2 ± 0.9 nm and an average length of around 65 nm (Figure 5d,f).

There was a periodic height fluctuation along the contour of the fibrils corresponding to the periodicity or pitch (Figure 5h). Long fibrils presented a periodic twist (pitch is defined as the distance between two consecutive peaks, *p*/2, along the contour length). The pitch size of the amyloid-like fibrils was estimated from AFM images using the autocorrelation function (ACF; Figure 5h). The system evolves toward a ∼50 nm pitch for long fibrils.

**Figure 6:**
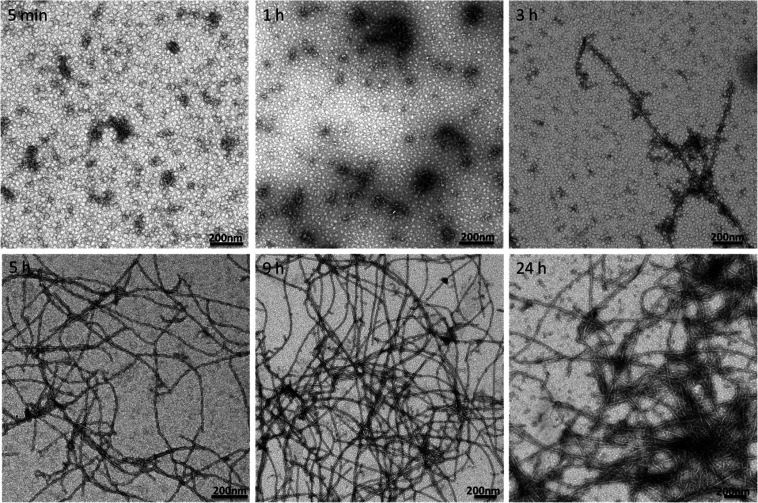
Stained TEM images of APO heated at 90 °C and pH 2 for different incubation times.

We also examined the morphology of growing fibrils by TEM analysis. Figure 6 shows images of APO fibrils acquired after different incubation times. In line with the previous AFM study (Figure 3), only small protein aggregates were formed after 1 h and long filaments only started to appear after 3 h. Again, the average contour length of filaments was seen to increase with incubation time.

**Figure 7:**
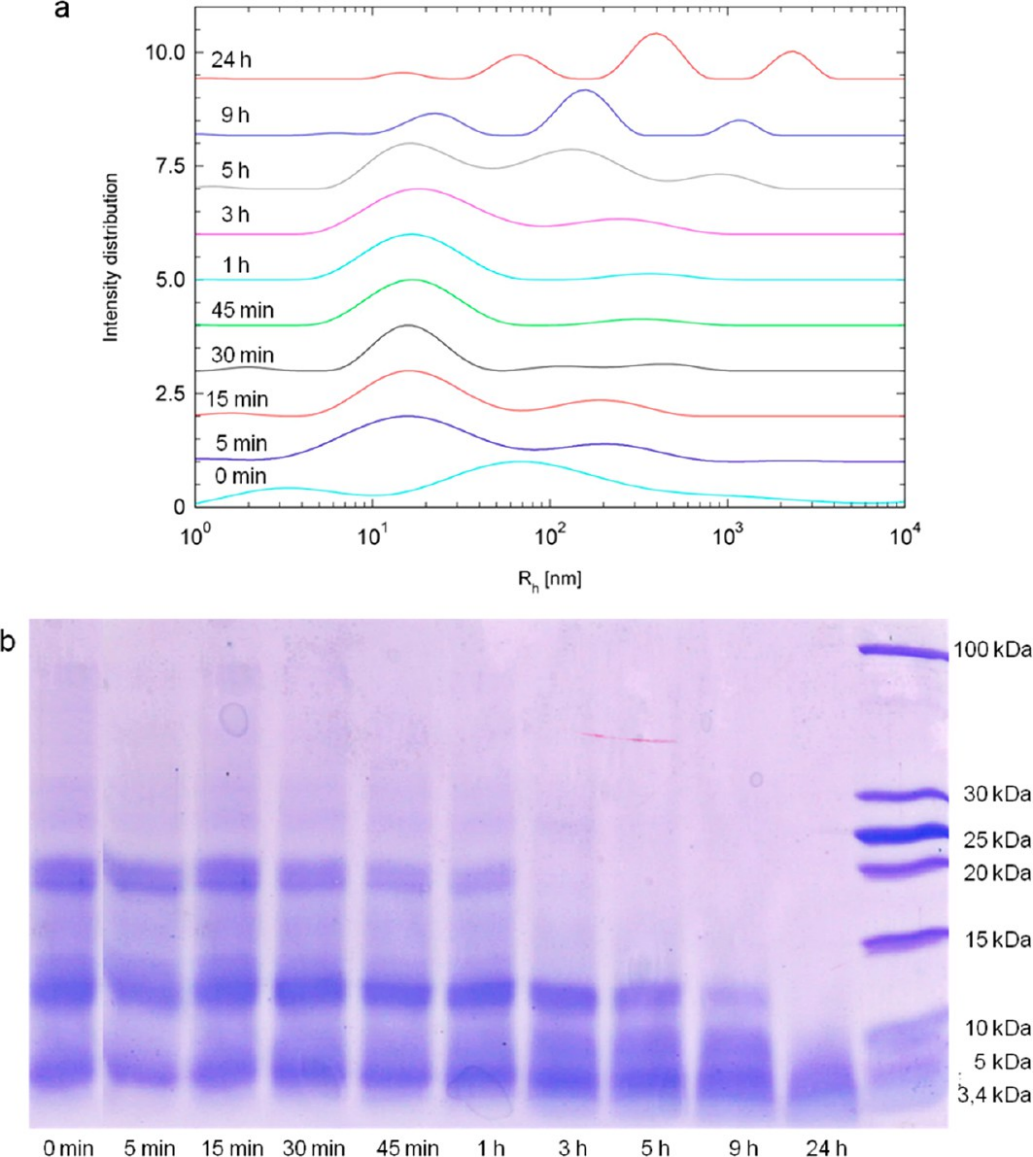
(a) Particle size distribution (hydrodynamic radius) of APO heated at 90 °C and pH 2 for different incubation times based on a CONTIN analysis of DLS data. (b) SDS-PAGE of APO heated at 90 °C and pH 2 for different incubation times.

Figure 7a shows the results of the CONTIN analysis from DLS experiments on the different time aliquots of the developing APO fibrils. Populations of larger aggregates developed with increasing incubation time. Between 0 and 5 min, that is, native, unassembled APO at pH 2, two population were evident: the first low-*M*_w_ peak centered around 3–5 nm may be assigned to small peptides precursors forming amyloid aggregates, related to the decrease in *M*_w_ due to hydrolysis, and a second one centered at around 100 nm due to the protein aggregating at this pH (Figure S1b). After 5 min heating, there were two different populations: one centered between 3–60 nm, in agreement with the initial oligomers/aggregates observed by AFM and TEM (Figures 3 and 6) and a second very broad peak suggesting that the aggregates are polydisperse. The particle size distribution was greater after 3 h, coinciding with the appearance of the first long fibrils. As incubation time increases, the number of protein oligomers decreases, and the populations associated with medium and long fibrils increase. At 9 h incubation, there were three different signals corresponding to the three different populations observed through AFM: small aggregates, medium fibrils, and long fibrils (Figure 3). After 24 h, the particle size population increased further, corresponding to the more aggregated fibrils seen with AFM. As a whole, the CONTIN analysis illustrates the conversion of the protein monomers into hydrolyzed peptides first, followed by larger aggregates.

We looked for the presence of peptide fragments at different incubation times using SDS-PAGE electrophoresis (Figure 7b) in a further attempt to determine whether they could be responsible for the formation of the amyloid aggregates observed in the present case. At pH 2, APO is dissociated into its 24 polypeptide subunits each with a molecular weight of around ∼20 kDa. The original APO subunit band at 20 kDa is still visible after 1 h of incubation, although the amount is already considerably reduced compared to the initial conditions. The appearance of the 11–13 kDa bands in the early stages of incubation is consistent with partial hydrolysis. A band at around 5 kDa was evident after incubation for 5 min, indicating a very high rate of hydrolysis. After 3 h of incubation, the native protein band at 20 kDa disappeared, which is compatible with the formation of medium and long rigid fibrils. After 24 h, the native APO protein was completely hydrolyzed and only low-molecular-weight peptide fragments (<5 kDa) were present in the system. These results confirm the decisive role of protein hydrolysis in the formation of amyloid fibrils.

**Figure 8:**
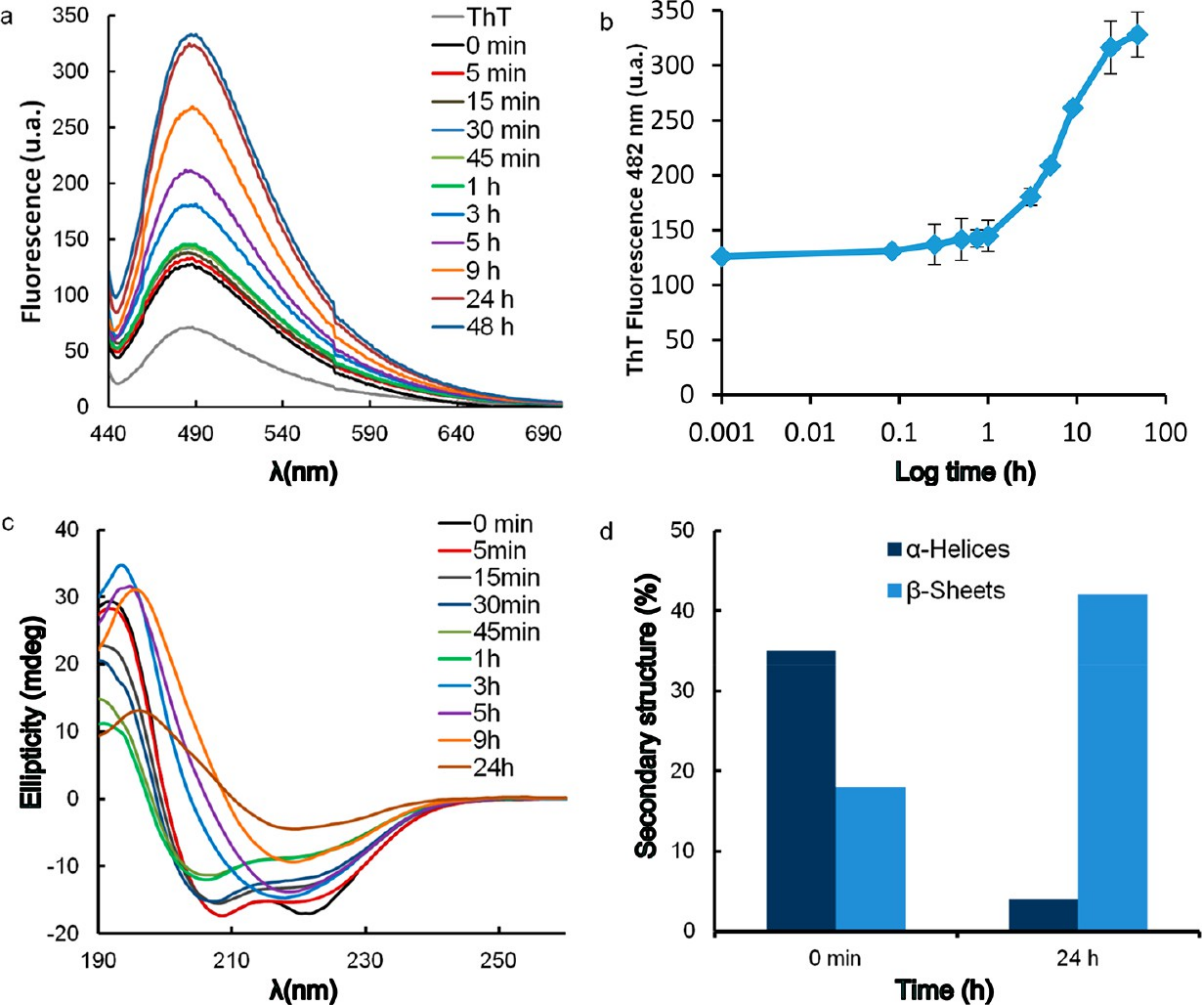
(a, b) Kinetics of APO fibril formation monitored by ThT fluorescence. (c) Far-UV circular dichroism of APO heated at 90 °C and pH 2 for different incubation times. (d) α-Helix and β-sheet percentages in APO at pH 2 without heating (0 min) and after incubation at 90 °C for 24 h.

We also studied the kinetics of APO aggregation by means of the thioflavin-T (ThT) fluorescence assay. The formation of amyloid aggregates was detected as an increase in ThT fluorescence intensity, with a characteristic emission band centered at 482 nm. Figure 8a shows the increase in ThT fluorescence upon binding to amyloid-like fibrils. In the early stages of aggregation, the fluorescence intensity increased, corresponding to the formation of small aggregates. There was a short lag phase in the first hour (Figure 8b) during which the fluorescence intensity remained low. At incubation times of over 3 h, when the first fibrils formed, the ThT fluorescence intensity increased and plateaued at a maximum after 24 h when mature fibrils were predominantly formed.

**Figure 9:**
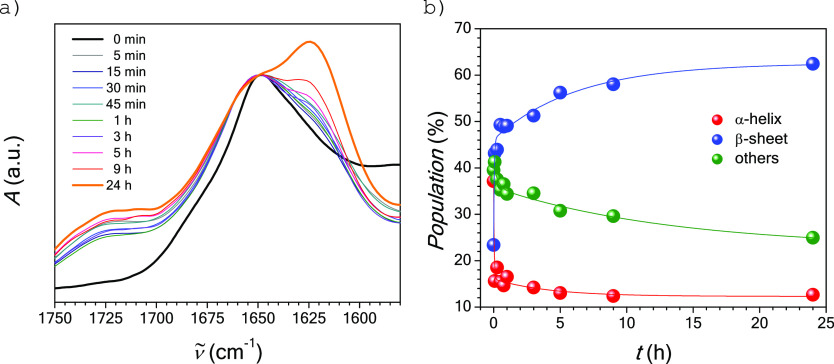
Evolution of (a) FTIR spectra and (b) percentages of secondary structures for APO heated at 90 °C and pH 2.

The hallmark of amyloid assemblies is a conformational transition of the constituent monomeric proteins into a β-sheet-rich fibril. Therefore, we followed changes in the secondary structure with incubation time using CD and FTIR (Figures 8c and 9a, respectively). The CD spectra initially obtained for samples containing long fibrils (3, 5, 9, and 24 h) were distorted (Figure S9); therefore, we had to remove the remaining nonfibrillar material and protein fragments from the fibril solutions through several centrifugation steps until the filtrate was protein-free according to UV–vis spectroscopy (see Materials and Methods and Figure S9).

The native APO protein incubated for 5 min shows a regular structure with a peak at 210 nm and a diffuse bump in the 220–230 nm region, both characteristic of the high α-helical content in the folded native protein (Figure 8c). A structural change had already occurred between 5 min and 1 h of incubation, and after 3 h there was a pronounced reduction in helical content relative to the initial spectrum, which is evident based on the decrease in ellipticity intensity over a wide range of wavelengths, consistent with the ThT observations. The sequence should be from α-helix to random (via denaturation and hydrolysis–physical and chemical damage) and slow assembly toward β-sheet formation in selected sequences. Between 3 and 24 h, the shoulder at around 217 nm suggests the presence of β-sheet structures, as expected for the observed well-ordered amyloid aggregates, and finally, the final CD spectra acquired at 24 h show the characteristic signature of β-sheet-rich amyloids. Figure 8d shows the CDPro software results for α-helix and β-sheet content determination in the aliquots sampled at 0 min and 24 h. Over time, the APO protein loses α-helix content and gains β-sheet structure, supporting a pathway toward amyloid fibril formation. The α-helix and β-sheet percentages for the different aliquots are shown in Table S2.

FTIR experiments provide further confirmation of the evolution of APO’s structure upon heating at 90 °C and pH 2 (Figure 9a). The initial native globular conformation clearly shows a main characteristic absorption peak at about 1655 cm^–1^, corresponding to the α-helical domains, which decreases during hydrolysis. On the other hand, an absorption peak at about 1623 cm^–1^, corresponding to the β-sheet domains characteristic of amyloid structures, starts to predominate over time. Deconvolution and peak analysis of the spectra (Figures S10 and Table S3) confirm the idea that the population of both α-helix domains and other secondary structures (turns and unordered structures) decreased from 37% to 12% and from 40% to 25%, respectively, during the incubation of APO for 24 h. These population reductions were accompanied by an increase in β-sheet population from 23% to 62% as shorter sequences (which self-assemble to create 1-D structures) were produced during the hydrolysis of APO protein–a chemical process evidenced by the increase of the absorption peak from 1700 to 1750 cm^–1^. Figure 9b shows the decreasing and increasing tendencies among the populations of secondary structures during the incubation of APO as a function of time

## Conclusions

We recently reported that ferritin, a key component in the regulation of brain iron homeostasis, forms amyloid fibrils that share common traits with the pathological amyloid fibrils found in Alzheimer’s and Parkinson’s disease. In this study, we optimized the chemical conditions to form long, rigid fibrils from an L-rich apoferritin, then described their fibril growth kinetics. An incubation period of 9 h, a protein concentration of 0.05–0.2 wt %, and a stirring rate of 90 rpm are the optimal parameters to form well-structured, long and semiflexible fibrils from APO protein.

We have demonstrated that the formation of apoferritin amyloid fibrils starts from short oligomer aggregates that develop into long, mature fibrils after 3 h at 90 °C. AFM statistical analysis, SDS-PAGE, CD, and FTIR all provide convincing evidence that protein unfolding and partial hydrolysis are essential for the formation of large aggregates, and that small peptide fragments (<5 kDa) are involved in fibril formation. AFM, TEM, and DLS measurements helped resolve and identify three different populations during the fibrillation process. All these results taken together deepen our understanding of apoferritin fibrillation kinetics.

## Supporting Information Available

Additional characterization: AFM images, statistical analysis, CD spectra before and after centrifuging aliquots, and estimation of protein secondary structure by CD and FTIR (PDF)
