# Supplementary material for: Understanding the Formation of Apoferritin Amyloid Fibrils
Source: Biomacromolecules. 2021 Apr 6;22(5):2057–66. doi: 10.1021/acs.biomac.1c00176 (PMC8462754; doi:10.1021/acs.biomac.1c00176)
Supplement: Supplementary file 1 — bm1c00176_si_001.pdf [file bm1c00176_si_001.pdf]

Supporting Information

Understanding the Formation of Apoferritin Amyloid Fibrils

Rocío Jurado,[†] Jozef Adamcik,[‡] Antoni Sánchez-Ferrer,[‡] Sreenath Bolisetty,[‡] Raffaele Mezzenga[‡,§] and Natividad Gálvez[†,]*

[†]Department of Inorganic Chemistry, University of Granada, 18071 Granada, Spain

[‡] Department of Health Sciences and Technology, ETH Zürich, 8092 Zürich, Switzerland

[§]Department of Materials, ETH Zürich, 8093 Zürich, Switzerland

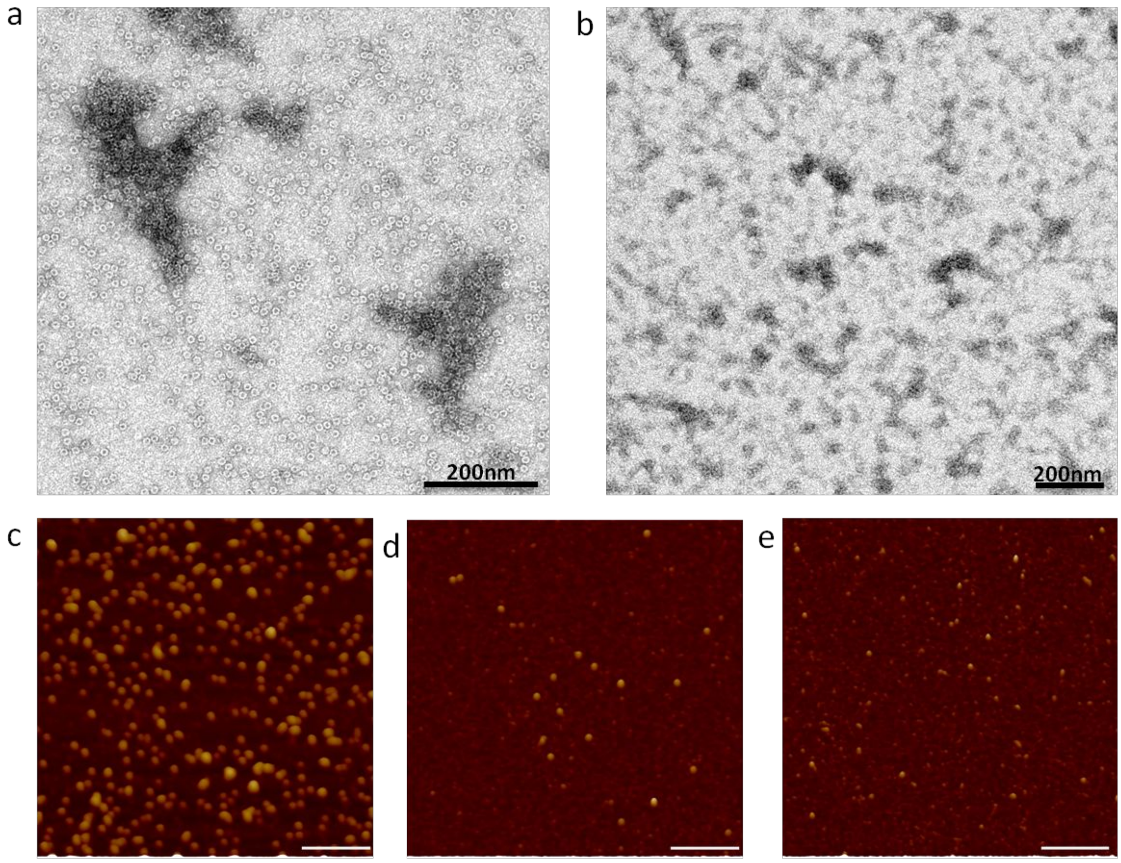

Figure S1. TEM images of (a) native APO at pH 7, and (b) at pH 2 after dilution. 3D AFM images of (c) native APO at pH 7, (d) at pH 2 after dilution, and (e) at pH 2 incubated for 120 h. Scale bars represent 200 nm.

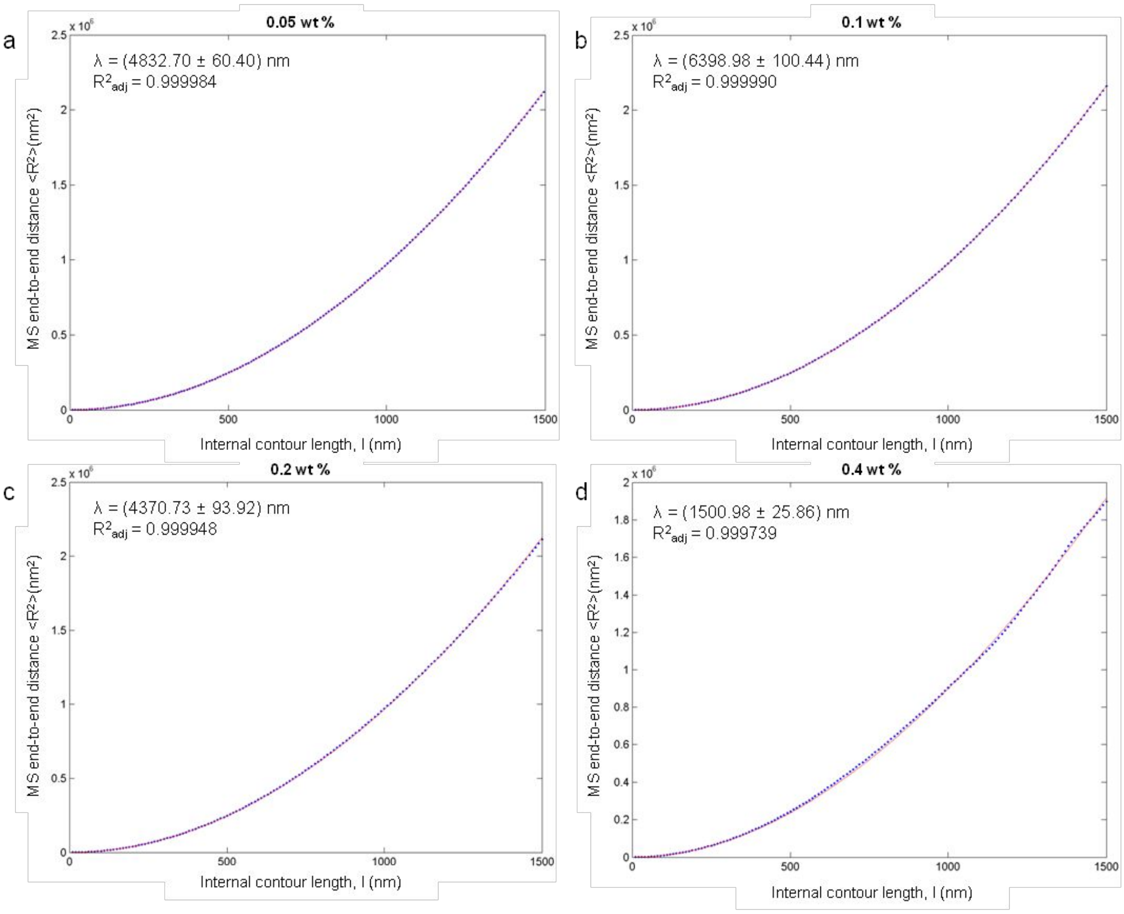

Figure S2. Calculated persistence length for (a) 0.05 wt.%, (b) 0.1 wt.%, (c) 0.2 wt.%, and (d) 0.4 wt.% APO after 9 h of incubation at 90 °C.

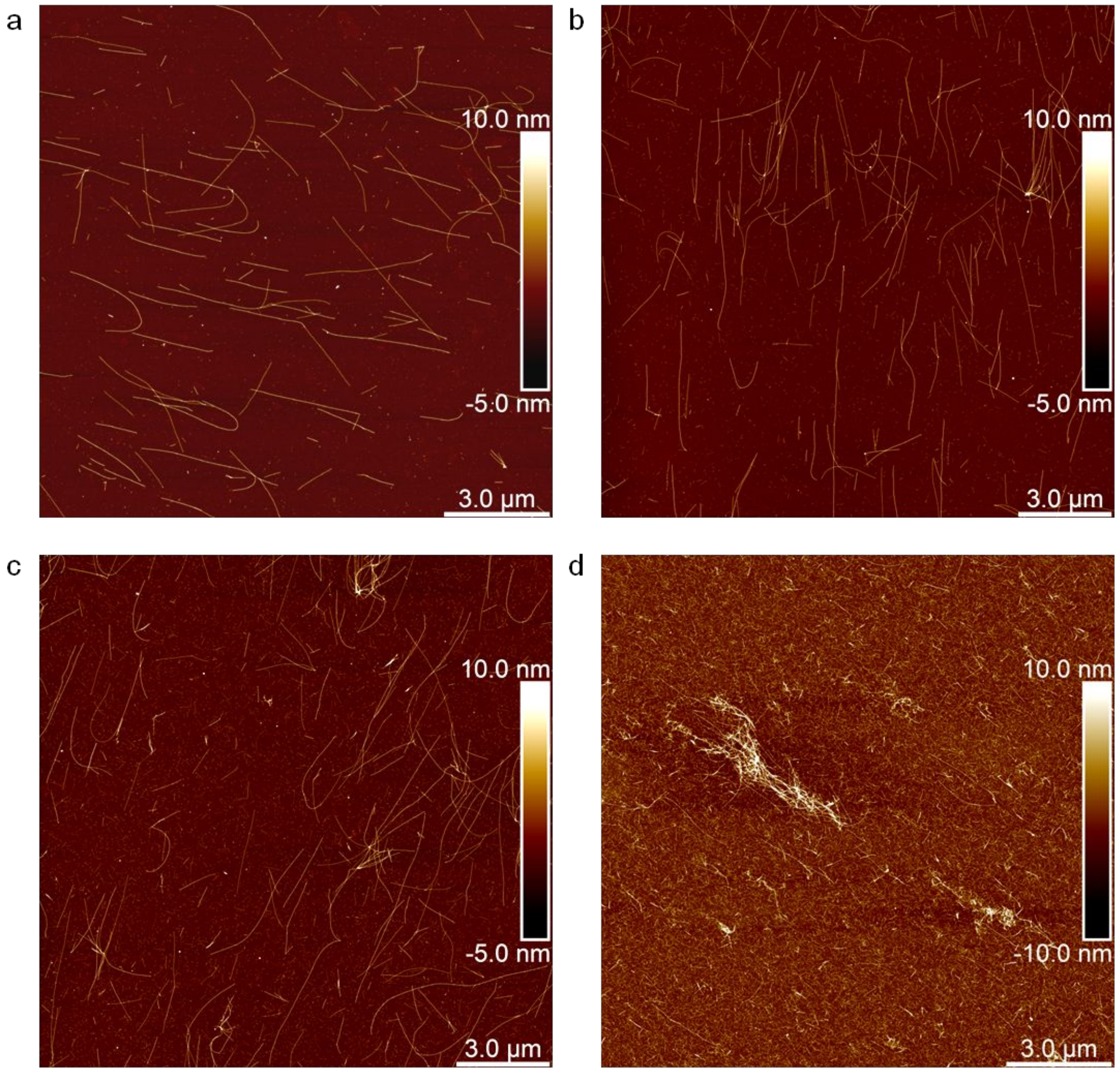

Figure S3. AFM images of (a) 0.05 wt %, (b) 0.1 % wt, (c) 0.2 wt %, and (d) 0.4 wt % APO heated at 90 °C after 9 h.

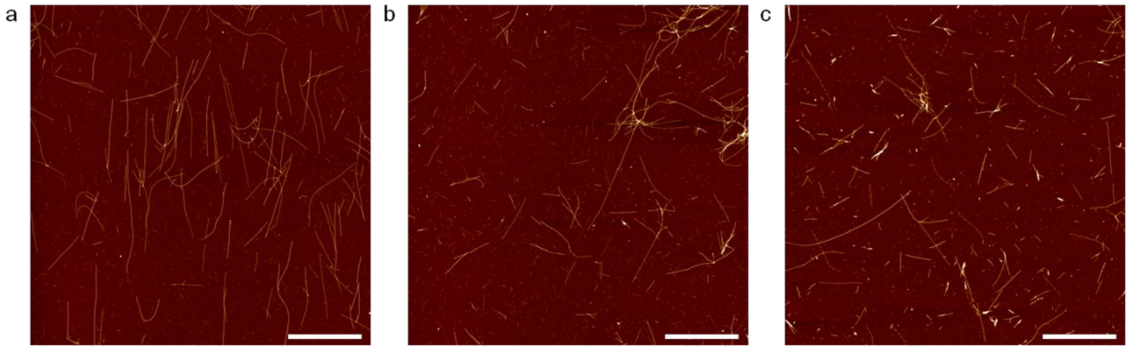

Figure S4. AFM images of APO heated at 90 °C (a) after 9 h at 90 rpm, (b) after 24 h at 90 rpm, and (c) after 9 h at 220 rpm. Scale bars represent 3 μm.

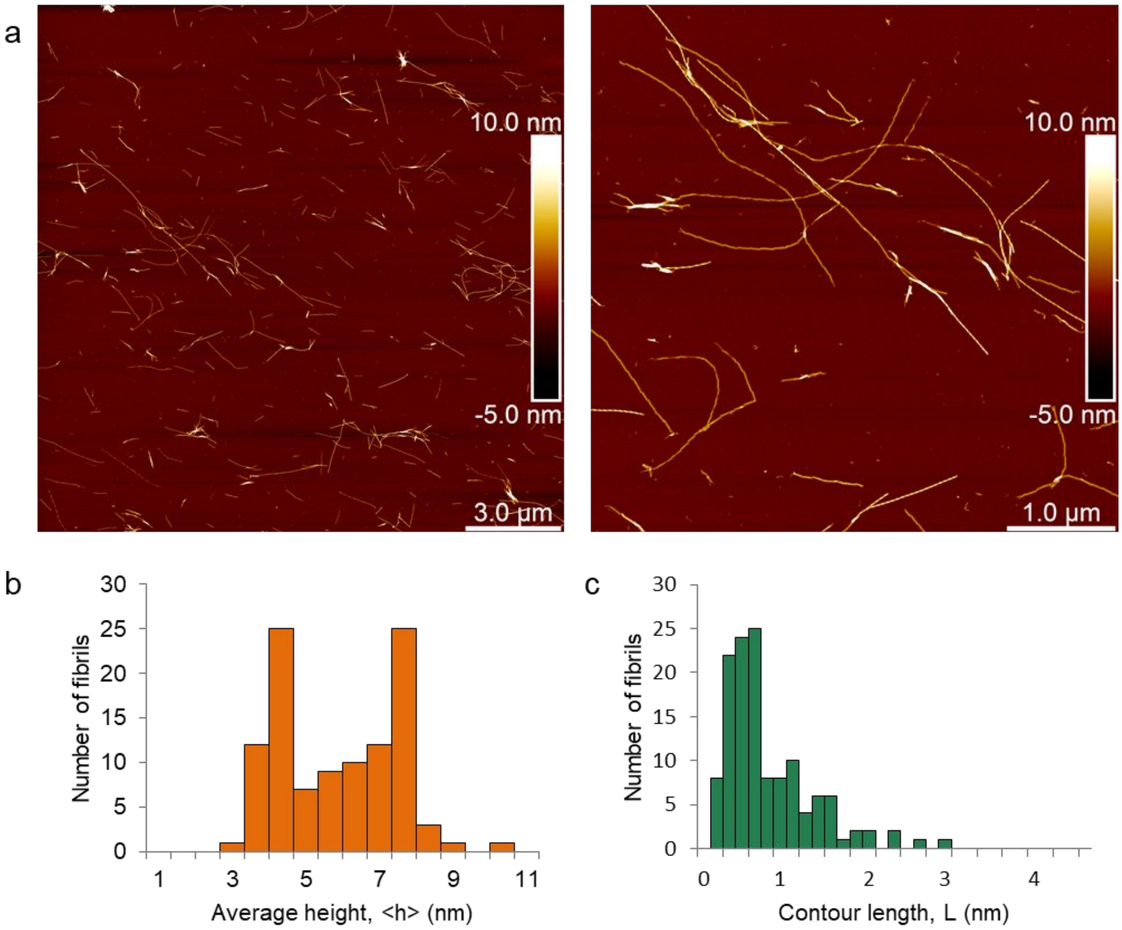

Figure S5. (a) AFM images, (b) average height distribution, and (c) contour length distribution of APO heated at 90 °C after 24 h at 220 rpm.

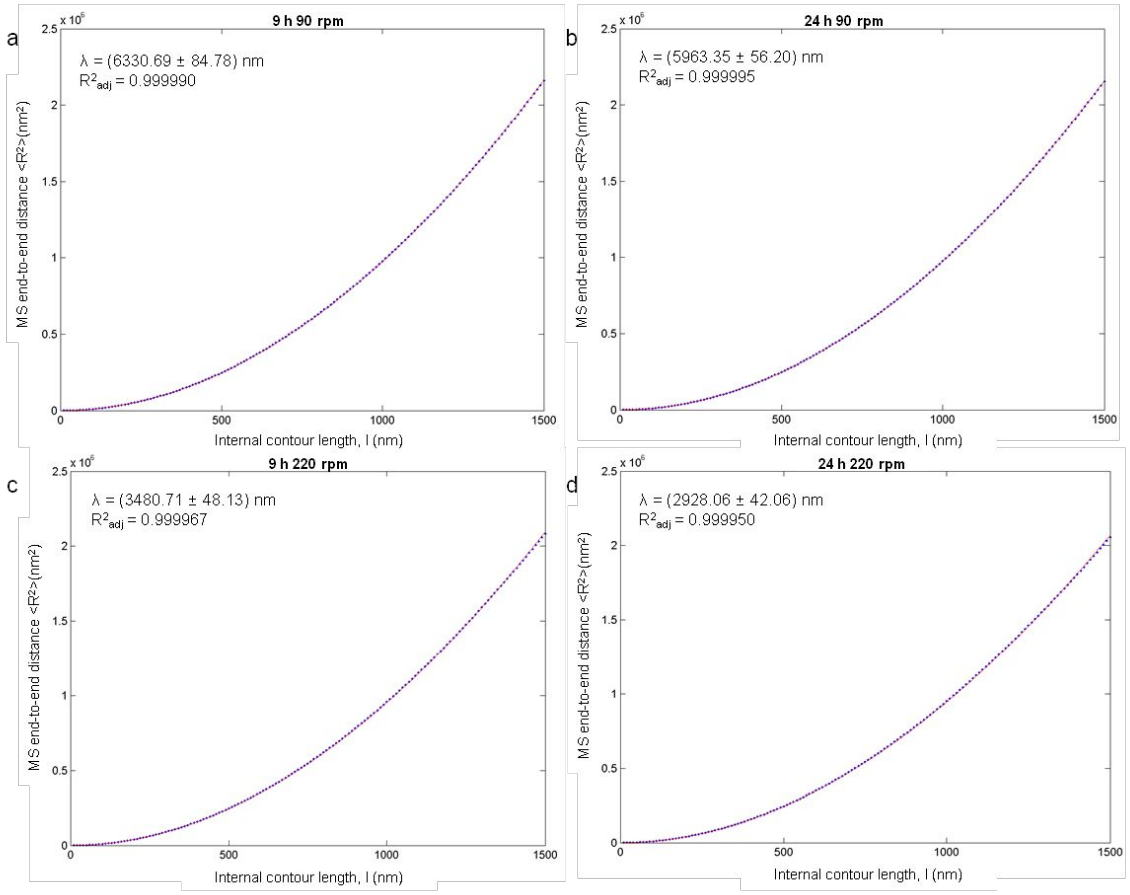

Figure S6. Calculated persistence length for APO heated at 90 °C: (a) after 9 h of incubation at 90 rpm, (b) after 24 h at 90 rpm, (c) after 9 h at 220 rpm, and (d) after 24 h at 220 rpm.

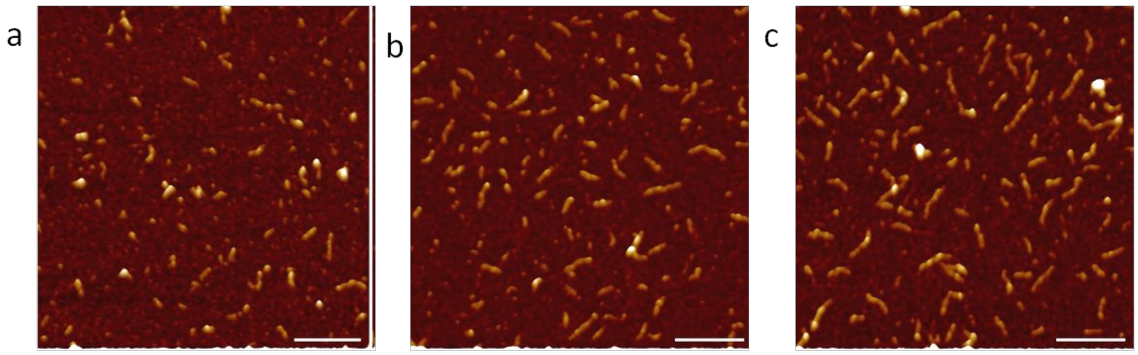

Figure S7. 3D AFM images of APO heated at 90 °C after (a) 5 min, (b) 45 min, and (c) 1 h. Scale bars represent 200 nm.

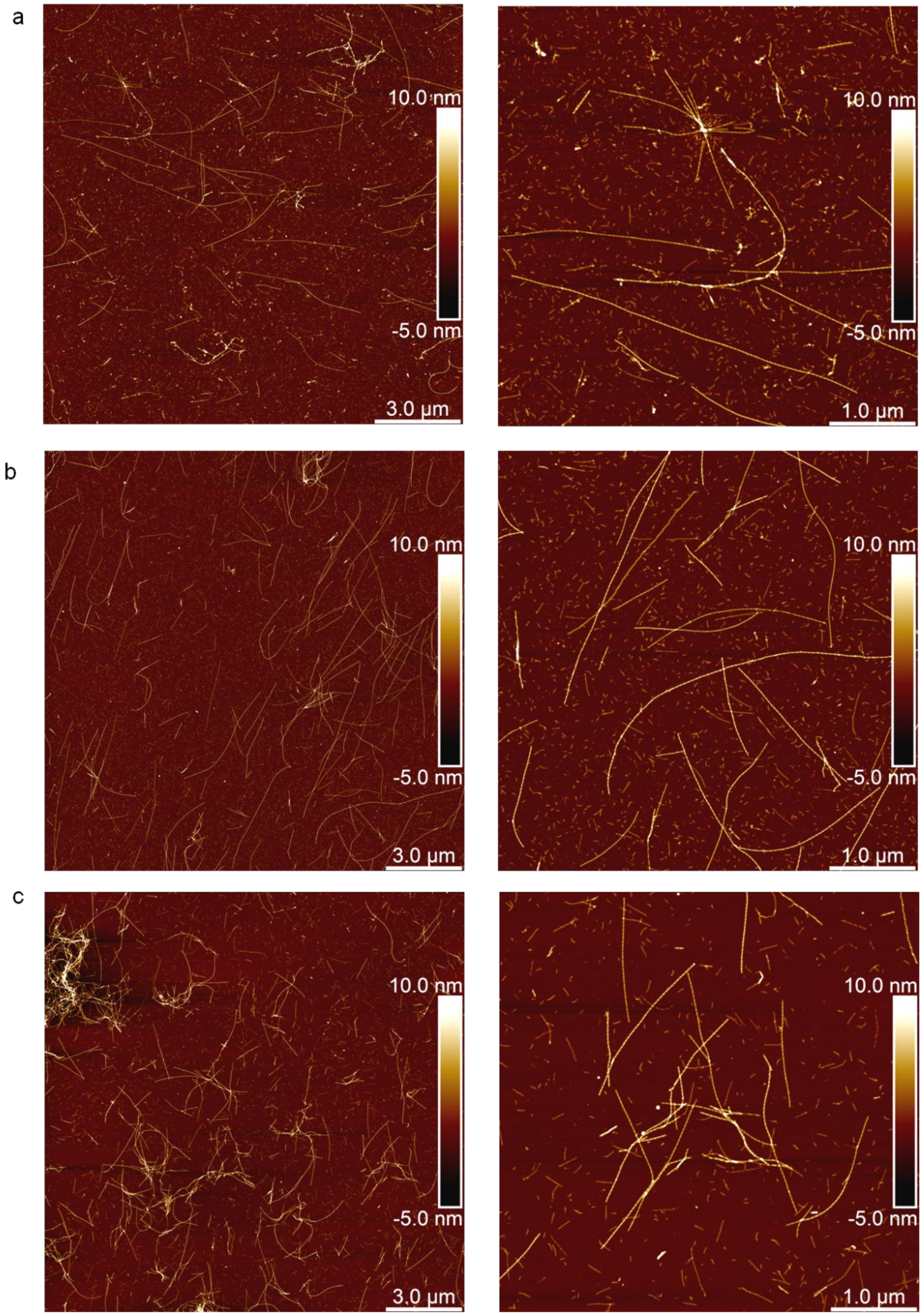

Figure S8. AFM images of APO heated at 90 °C after (a) 5 h, (b) 9 h, and (c) 24 h. It can be observed small and medium

aggregates coexisting together with long fibrils in the three cases.

	Small aggregates			Long fibrils			
Time	N	Height	Length	N	Height	Length	Pitch
3h	500	2,75 ± 0,88	72,05 ± 24,2	57	5,28 ± 1,29	1631,4 ± 1216,8	53,7
5 h	500	3,49 ± 0,88	68,45 ± 19,8	92	6,89 ± 1,34	1573,83 ± 1174,1	52,1
9 h	500	3,80 ± 0,98	49,48 ± 15,2	257	6,92 ± 1,24	1888,33 ± 1346,1	47,8
24 h	500	3,06 ±1,04	67,54 ± 28,9	628	6,57 ± 1,60	1013,23 ± 756,9	47,8

Table S1. Basic structural information about APO small aggregates and long fibrils at diferent incubation times. N=number of filaments tracked. All measurements are expressed in nanometers.

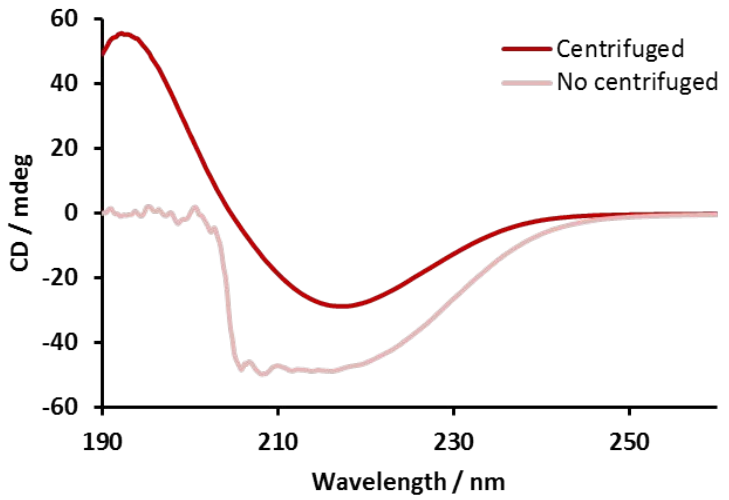

Figure S9. Far-UV circular dichroism of APO fibrils heated for 3 h, before and after being centrifuged.

Time	α-helix	Strand	turns	unordered
**0 min**	35.1	18.6	18.4	27.9
**5min**	15.4	31.9	21.4	31.2
**15min**	8.5	37.7	21.1	32.7
**30min**	7.8	40.0	20.8	31.4
**45min**	6.0	42.6	21.4	30.0
**1h**	6.0	42.2	21.4	32.1
**3h**	5.7	39.3	22.4	32.6
**5h**	4.6	40.6	22.2	32.6
**9h**	3.7	41.9	21.8	32.4
**24h**	3.6	41.7	21.4	33.3

Table S2. Estimation of protein secondary structure of APO after different incubation times at pH 2 and 90 °C from the CD analysis. The content is expressed in %.

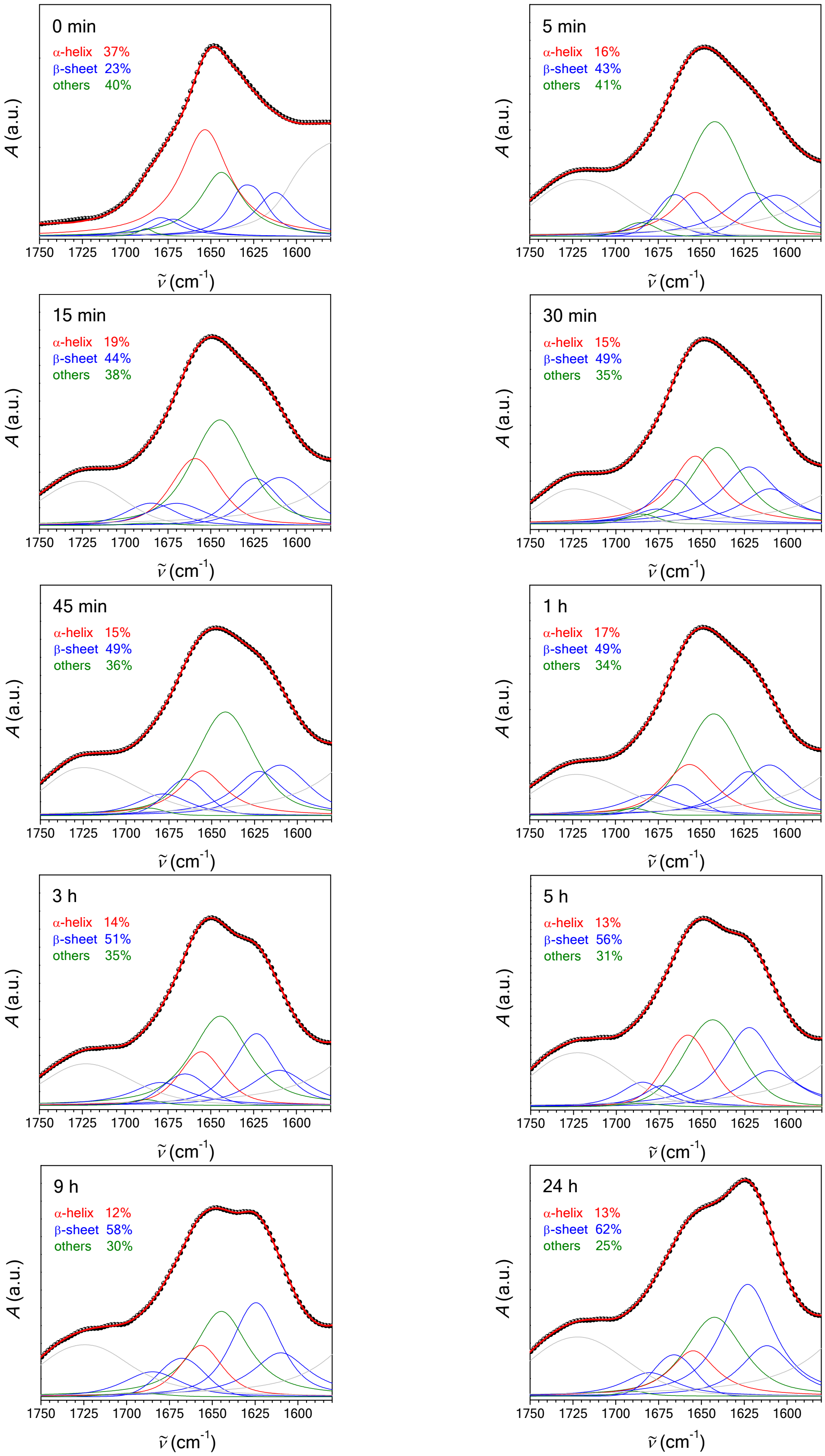

0 min
α-helix 37%
β-sheet 23%
others 40%
5 min
α-helix 16%
β-sheet 43%
others 41%
15 min
α-helix 19%
β-sheet 44%
others 38%
30 min
α-helix 15%
β-sheet 49%
others 35%
45 min
α-helix 15%
β-sheet 49%
others 36%
1 h
α-helix 17%
β-sheet 49%
others 34%
3 h
α-helix 14%
β-sheet 51%
others 35%
5 h
α-helix 13%
β-sheet 56%
others 31%
9 h
α-helix 12%
β-sheet 58%
others 30%
24 h
α-helix 13%
β-sheet 62%
others 25%
A (a.u.)
ṽ (cm-1)
1750 1725 1700 1675 1650 1625 1600

Figure S10. FTIR spectra with the corresponding deconvolution analysis for the APO heated at 90 °C and pH 2 at different incubation times.

time	α-helix (%)	β-sheet (%)	random (%)
0	37.1	23.4	39.5
5 min	15.6	43.1	41.3
15 min	18.5	43.9	37.5
30 min	15.4	49.3	35.3
45 min	14.6	48.9	36.5
1 h	16.5	49.1	34.4
3 h	14.2	51.3	34.5
5 h	13.0	56.2	30.8
9 h	12.4	58.0	29.6
24 h	12.6	62.4	24.9

Table S3. Estimation of the protein secondary structure of APO after different incubation times at 90 °C and pH 2 from the FTIR analysis.
